# TMFUF: a triple matrix factorization-based unified framework for predicting comprehensive drug-drug interactions of new drugs

**DOI:** 10.1186/s12859-018-2379-8

**Published:** 2018-11-20

**Authors:** Jian-Yu Shi, Hua Huang, Jia-Xin Li, Peng Lei, Yan-Ning Zhang, Kai Dong, Siu-Ming Yiu

**Affiliations:** 1 School of Life Sciences, Northwestern Polytechnical University, Xi’an, China; 2 School of Software and Microelectronics, Northwestern Polytechnical University, Xi’an, China; 3 Department of Chinese Medicine, Shaanxi Provincial People’s Hospital, Xi’an, China; 4 School of Computer Science, Northwestern Polytechnical University, Xi’an, China; 5 Department of Computer Science, the University of Hong Kong, Hong Kong, China

**Keywords:** Drug-drug interaction, Side effects, Matrix factorization, Prediction, Regression

## Abstract

**Background:**

A significant number of adverse drug reactions is caused by unexpected Drug-drug interactions (DDIs). The identification of DDIs becomes crucial before the co-prescription of multiple drugs is made. Such a task in clinics or in drug discovery usually requires high costs and numerous limitations, while computational approaches are able to predict potential DDIs effectively by utilizing diverse drug attributes (e.g. side effects). Nevertheless, they’re incapable when required to predict enhancive and degressive DDIs, which change increasingly and decreasingly the pharmacological behavior of interacting drugs respectively. The pharmacological change of DDIs is one of the most important factors when making a multi-drug prescription.

**Results:**

In this work, we design a Triple Matrix Factorization-based Unified Framework (TMFUF) to address the above issue. By leveraging a group of side effect entries of drugs, TMFUF achieves the inspiring result (AUC = 0.842 and AUPR = 0.526) in the case of conventional DDI prediction under the traditional screening task. In the comparison with two state-of-the-art approaches, TMFUF demonstrates it superiority by ~ 7% and ~ 20% improvement in terms of AUC and AUPR respectively. More importantly, TMFUF shows its ability in the comprehensive DDI prediction under different screening tasks. Finally, a utilization TMFUF reveals the significant pairs of side effects, which contribute to form enhancive and degressive DDIs, for further clinical validation.

**Conclusions:**

The proposed TMFUF is first capable to predict both conventional binary DDIs and comprehensive DDIs such that it captures the pharmacological changes caused by DDIs. Furthermore, it provides a unified solution of DDI prediction for two screening scenarios, which involves newly given drugs having no prior interaction. Another advantage is its ability to indicate how significantly the pairs of drug features contribute to form DDIs.

## Background

Two or more drugs in a joint prescription would influence each other in terms of pharmacological behavior [1]. This kind of influence, termed as Drug-Drug Interaction (DDI), could reduce efficacy, induce unexpected toxicities or other adverse drug reactions. Unidentified DDIs would generate unsafe treatments and even medication errors for those patients under the treatment with multi-drug medications [2–5].

Since the number of unidentified DDIs is nearly proportional to the number of newly approved drugs to the power of 2, the broadcasting of DDI-induced adverse effects across medications cannot be negligible. Therefore, DDI identification becomes an urgent need before clinical medications are administered. However, traditionally based on cytochrome P450 testing [6] or transporter-associated interactions [7], the approaches in clinical trials have usually a burden of high cost, long duration and even animal welfare considerations [8], and also face unavoidable challenges, such as inadequate participants and numerous drug pairs to be screened. Consequently, a few DDIs can be identified during drug discovery and development, while most of them are reported in clinics after the corresponding drugs enter the market.

Computational approaches have been developing as a promising assistant of biological/chemical experiments. Both pharmacological research and pharmacy companies pay more attention to them recently [9, 10], because they can rapidly infer potential DDIs among a large number of drug pairs. Current computational approaches can be roughly grouped as text mining-based and machine learning-based approaches. The former detects approved DDIs from diverse text sources [8], such as scientific literature [11, 12], the Adverse Event Reporting System of FDA (http://www.fda.gov) and electronic medical records [13]. However, these approaches largely depend on the post-market evidence, such that they cannot alert potential DDIs before multi-drug prescriptions are made. In contrast, the latter can provide such an alert by leveraging the techniques of machine learning (e.g. network recommendation-based [8], Naïve similarity-based approach [14], classification-based [15]). These approaches extract drug features or similarities based on diverse pre-marketed drug properties, such as chemical structures [14], hierarchical classification codes [15], targets [16] and side effects [8]. To the best of our knowledge, the great majority of existing machine learning-based approaches are only able to predict how likely two drugs interact with each other (named as conventional binary prediction). However, two interacting drugs definitely influence each other in terms of pharmacological response in vivo.

It is more significant to identify whether DDIs increase or decrease the behaviors of the interacting drugs in many cases, such as optimizing patient care, establishing drug dosages and finding drug resistance in multi-drug treatments [17]. For two drugs interacting with each other, the occurrence of their interaction would increase or decrease their serum concentration, which is the pharmacological index of measuring the amount of a drug in the pharmacokinetic circulation [18]. For example, when Cyclosporine (who’s DrugBank Id is DB00091) is taken with Ticagrelor (DB08816) together, their interaction would increase their serum concentration. While taken with Vincristine (DB00541), the interaction would decrease their serum concentration. Briefly, the first case of DDI is named as an enhancive DDI and the second one is termed as a degressive DDI in the following texts.

In summary, the existing machine learning-based approaches were developed for conventional binary DDIs only, but not for enhancive and degressive DDIs. Moreover, although these approaches can predict the interactions between the drugs having known DDIs and the new drugs having none of existing DDI, they cannot predict the interactions among new drugs. This prediction task is important and helpful to reveal the underlying mechanism of forming DDIs [19].

To address abovementioned issues, this work proposes a Unified Framework of DDI Prediction based on Triple Matrix Factorization (TMFUF). The remaining texts are organized as follows. Section Materials and Method introduces the collection of comprehensive DDIs, the problems of both conventional and comprehensive DDI prediction, the design of our TMFUF, and appropriate cross-validation schemes with respect to different screening scenarios. Section Experiment describes the preparation of DDI prediction, comparison with former approaches in the conventional DDI prediction, and predicting performance of TMFUF under different screening scenarios for both binary and comprehensive prediction. Finally, the last section draws our conclusion.

## Methods

### Dataset

After searching DrugBank [20], we first acquired 2329 approved small-molecular drugs. Among them, we then removed a set of drugs, which have no DDI entry in DrugBank or have no off-label side effect record in OFFSIDES [21]. Last, we obtained a set of 603 drugs, of which each drug has at least one DDI and at least one off-label side effect record. Totally, the DDIs among those 603 drugs contains 24,114 DDIs, including 18,710 enhancive DDIs (EnI) and 5404 degressive DDIs (DeI). Moreover, the set of side effects with regard to those 603 drugs finally contains 9149 unique side effect entries, such that each drug can be encoded into a 9149-dimensional feature vector. See also the next section for technical details.

Moreover, we represent these DDIs as a network, of which nodes are drugs and edges are DDI and then summarize the fundamental properties of the DDI network (Table 1).

**Table 1:** Statistics of comprehensive DDI dataset

Property	Entries	Value
Global	Number of Drugs	603
	Number of Interactions	24,114
	Number of EnI	18,710
	Number of DeI	5404
Drug Degree	Average Degree of Drug	79.98
	Median Degree of Drug	65
	Max. Degree of Drug	310
	Min. Degree of Drug	1
Drug EnI Degree	Average EnI Degree	62.06
	Median EnI Degree	47
	Max. EnI Degree	242
	Min. EnI Degree	0
Drug DeI Degree	Average DeI Degree	17.92
	Median DeI Degree	9
	Max DeI Degree	219
	Min DeI Degree	0

### Problem formulation of DDI prediction

Denote **D** = {*d*_*i*_}, *i* = 1, 2, …, *m* as a set of *m* approved drugs which have known DDIs, and **D**_*u*_ = {*d*_*j*_}, *j* = 1, 2, …, *n* as n newly-given drugs, which have no prior DDIs. The interactions among *m* approved drugs are organized into an *m* × *m* symmetric interaction matrix **A**_*m* × *m*_ = {*a*_*ij*_}. From the point of view of graph theory, it is the adjacent matrix of a DDI network. In a network of conventional binary DDIs, *a*_*ij*_ ∈ {0, 1}, where *a*_*ij*_ = 1 if *d*_*i*_ interacts with *d*_*j*_, and *a*_*ij*_ = 0 otherwise. For the comprehensive DDI, *a*_*ij*_ ∈ {−1, 0, +1}, where *a*_*ij*_ =  + 1 indicates an enhancive DDI, *a*_*ij*_ =  − 1 indicates a degressive DDI, and *a*_*ij*_ = 0 indicates no interaction between *d*_*i*_ and *d*_*j*_ respectively. The conventional binary DDI matrix **A**_*b*_ is just a special case of the comprehensive DDI matrix **A** since it can be generated by **A**_*b*_ = *Binary*(**A**).

Side effects are selected to characterize these drugs, including the approved drugs and the newly-given drugs. According to the clinical occurrence of side effects, each drug *d*_*i*_ is characterized by a high-dimensional feature vector **f**_*i*_ = [*f*_1_, *f*_2_, …, *f*_*k*_, …, *f*_*p*_], where *f*_*k*_ = 1  or  0 indicates whether the *k*-th specific entry of side effect is observed or not. The feature vectors of the drugs in **D** and **D**_*u*_ are sequentially stacked as an *m* × *p* feature matrix **F**_*m* × *p*_ and an *n* × *p* feature matrix **F**_*n* × *p*_ respectively.

**Fig. 1:**
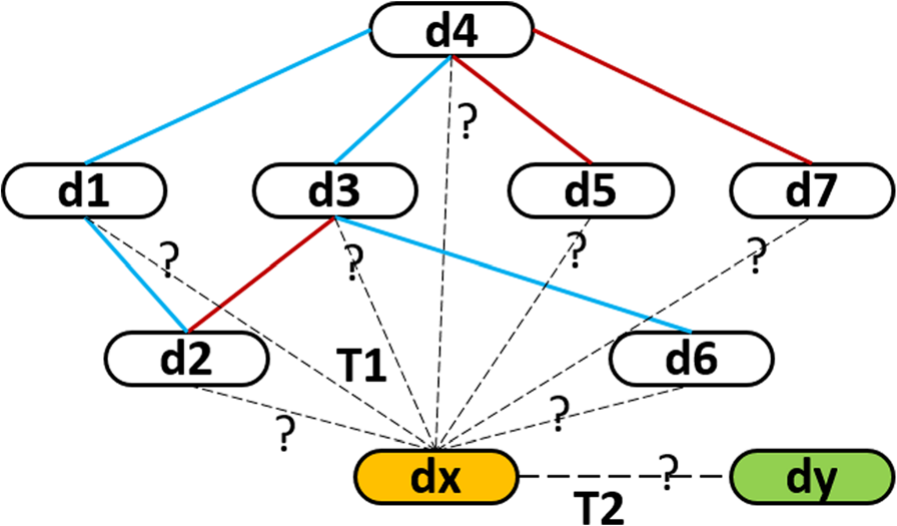
Illustration of predicting tasks. Drugs in DDI network are represented as capsule-like nodes. Known drugs are numbered from d1 to d7 and their interactions are denoted by the solid lines. Enhancive interactions and degressive interactions are highlighted by red lines and blue lines respectively. Two newly given drugs are the isolated nodes, which are labeled by dx and dy, and are filled by yellow and green respectively. Two types of predicting tasks, tagged by ‘T1’ and ‘T2’, are indicated by dotted lines. T1 predicts how likely dx interacts with those known drugs, while T2 predicts how likely dx interacts with dy. Both of them predict whether these potential interactions increase or decrease the pharmacological behaviors of these interacting drugs

For short, **a known/approved drug** is referred to as the drug in **D** and **a new drug** is referred to as the drug, which has no known interaction. This paper considers on two screening scenarios involving new drugs (Fig. 1). Their corresponding predicting tasks are defined as follows. The first task (T_1_) infers the potential interactions between known drugs and new drugs (e.g. d4 and dx in Fig. 1) while the second one (T_2_) infers the potential interactions among new drugs (e.g. dx and dy in Fig. 1). Both of them are required to predict how likely the potential interactions involving new drugs are enhancive or degressive.

### Triple matrix factorization and unified predicting model

Since new drugs are isolated nodes in the DDI network (Fig. 1), we cannot deduce their potential interactions by only their topological information. Thus, their additional information (e.g. side effects) is needed. We first extract features based on it and then train a supervised model of DDI prediction.

The underlying idea of the proposed model is to connect the features of known drugs in D with their DDI network topology in a certain way. We model such a connection as a bi-linear regression, which can be represented as a triple matrix factorization (TMF),

1 $$ \mathbf{A}=\mathbf{F}\boldsymbol{\Theta } {\mathbf{F}}^{\prime }, $$

where **A** is the adjacent matrix of a DDI network, **F** denotes the feature matrix of the drugs, and the symmetric projection matrix **Θ** plays a role of connecting the features of drugs to the interactions between drugs. Each entry *θ*_*rs*_ in **Θ** indicates how well the drug feature pair (*f*_*r*_, *f*_*s*_) contributes to form an enhancive or a degressive interaction. To simplify mathematical symbols in formulas, the apostrophe on “**F**” in Formula (1) indicates the transpose operator on it. The subsequent formulas in this paper adopt the same notation.

Nevertheless, Formula (1) cannot be solved by **Θ** = (**F**)^−1^**A**(**F**^′^)^−1^ directly because of three aspects. First, the inverse matrix of **F** doesn’t exist when *p* ≫ *m*. Secondly, there exists the multi-collinearity between the columns in **F** because the feature entries may not be independent to each other. Thirdly, it is rare to meet an **A** of full-rank. Moreover, we may solve it directly by ${\boldsymbol{\Theta}}^{\ast }=\arg \min \kern0.5em {\left\Vert \mathbf{A}-{\mathbf{F}}_{\mathbf{d}}\boldsymbol{\Theta} {\mathbf{F}}_{\mathbf{d}}^{\prime}\right\Vert}^2$. However, it is very difficult to estimate *p* × *p* entries in **Θ** when *p* is large (here *p* = 9149). Thus, we propose a new approach to solve **Θ**.

We suppose that there exists a low-dimensional latent feature space (interaction space), in which each drug is represented by a latent feature vector and the inner products between drugs are positively correlated with their interactivity. In other words, two drugs possibly form an interaction if they are near to each other and vice versa. Moreover, we also assume that the latent features of the drugs are related to their observed features of drugs. Therefore, the solution of **Θ** can be achieved as follows

2 $$ {\displaystyle \begin{array}{l}{\mathbf{A}}_{\mathbf{d}}^{\ast }=\arg \min {\left\Vert \mathbf{A}-{\mathbf{A}}_{\mathbf{d}}{\mathbf{A}}_{\mathbf{d}}^{\prime}\right\Vert}^2\\ {}{\mathbf{B}}^{\ast }=\arg \min \kern0.5em {\left\Vert {\mathbf{A}}_{\mathbf{d}}^{\ast }-\mathbf{FB}\right\Vert}^2,\\ {}{\boldsymbol{\Theta}}^{\ast }={\mathbf{B}}^{\ast }{\left({\mathbf{B}}^{\ast}\right)}^{\prime}\end{array}} $$

where the first item in the formula reflects the latent space by matrix factorization, the second one builds the bridge between the observed feature space and the latent feature space of drugs. In detail, **A**_**d**_ is the *m* × *r* latent interaction matrix, of which each row represents the feature vector of a drug in the latent space. Considering the symmetry of **A**, we obtain **A**_**d**_ by singular value decomposition (SVD) as follows,

3 $$ \mathbf{A}=\mathbf{U}\boldsymbol{\Sigma } {\mathbf{U}}^{\prime }=\mathbf{U}\sqrt{\boldsymbol{\Sigma}}\sqrt{{\boldsymbol{\Sigma}}^{\prime }}{\mathbf{U}}^{\prime }={\mathbf{A}}_{\mathbf{d}}{\mathbf{A}}_{\mathbf{d}}^{\prime }. $$

The *p* × *r* regression coefficient matrix,**B**, accounts for the regression between **A**_**d **_and **F**. The parameter *r* denotes **A**'s rank, which satisfies *r* < *m* and reflects the topological complexity of DDI network. So far, we only need to estimate *p* × *r* entries in **B** but not *p* × *p* entries in **Θ**. Partial Least Square Regression is adopted to solve B, because of *m* ≪ *p* and the multi-collinearity between feature columns (see also the next section).

**Fig. 2:**
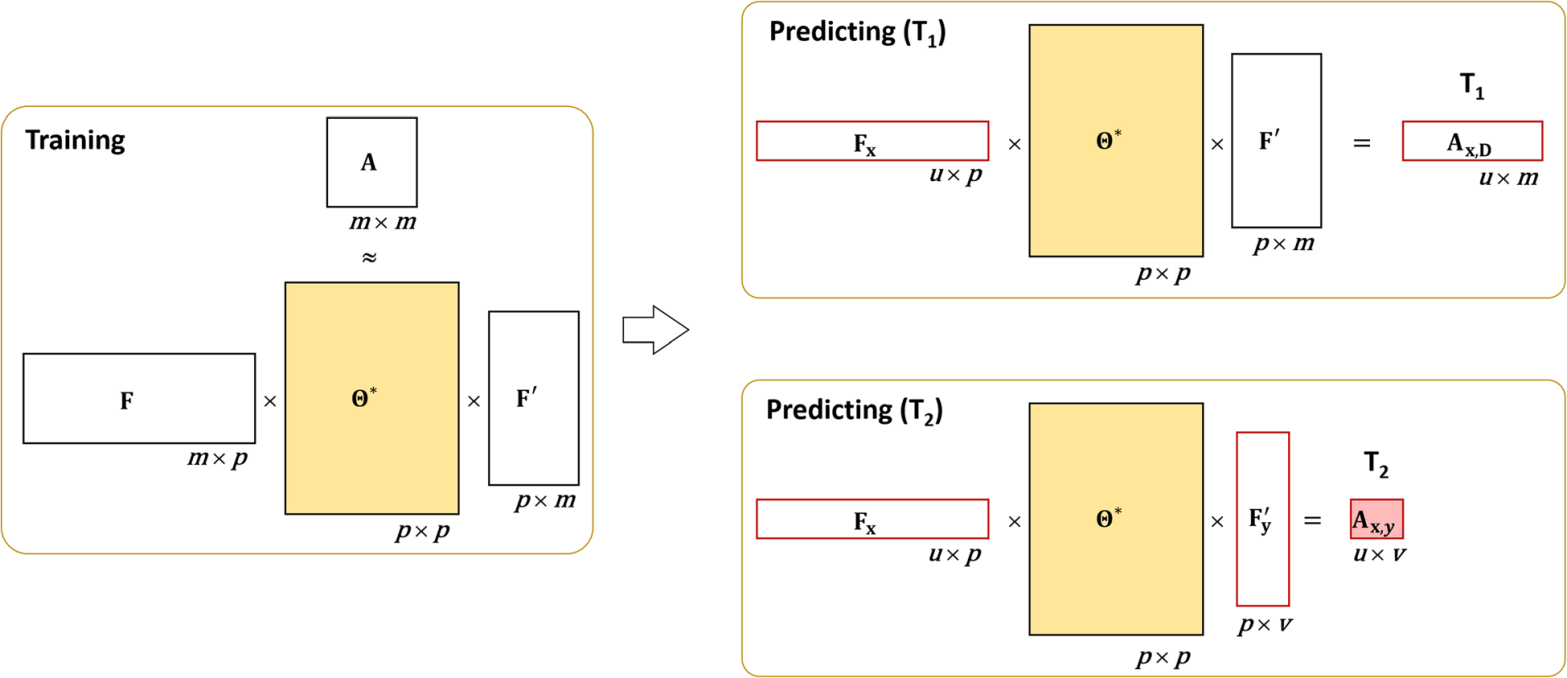
Supervised model of TMFUF. In the training phase, both A and F are used to calculate the symmetric projection matrix **Θ**. In the predicting model, the feature vectors and the projection matrix **Θ** are used to deduce potential DDIs for T_1_ and T_2_

After solving **Θ**^∗^, we derive the predicting model for both T_1_ and T_2_ from TMF in a Unified Framework (TMFUF) as follows.

4 $$ {\mathbf{A}}_{\mathbf{x},\mathbf{D}}={\mathbf{F}}_{\mathbf{x}}{\boldsymbol{\Theta}}^{\ast }{\mathbf{F}}^{\prime },{\mathbf{A}}_{\mathbf{x},\mathbf{y}}={\mathbf{F}}_{\mathbf{x}}{\boldsymbol{\Theta}}^{\ast }{\mathbf{F}}_{\mathbf{y}}^{\prime }, $$

where **F** is the *m* × *p* matrix feature matrix of known drugs, **F**_**x**_ and **F**_**y**_ are the *u* × *p* feature matrix of *u* new drugs {*d*_*x*_} and the *v* × *p* feature matrix of *v* new drugs {*d*_*y*_} respectively. In addition, **A**_**x**,**D**_ is the *u* × *m* confidence matrix, of which each entry *a*_*x*, *i*_ indicates how possibly a new drug *d*_*x*_ interacts with a known drug *d*_*i*_ ∈ **D**. **A**_**x**,**y**_ is the *u* × *v* confidence matrix, of which each entry *a*_*x*, *y*_ indicates how possibly *d*_*x*_ interacts with another new drug *d*_*y*_. The signs of the entries in both of two confidence matrices indicate types of comprehensive DDIs, enhancive or degressive respectively. Their absolute values are just confidences scores. The larger they are, the more likely they are interactions. Both the training phase and the predicting model are illustrated in Fig. 2.

### Partial Least Square regression

Linear regression can detect the linear relations between two groups of variables (the predictor **X**_*m* × *p*_ and the response **Y**_*m* × *r*_) with respect to *m* observations. In this context, we regard drugs as observations, **F** as their predictor matrix and **A**_**d**_ as their response matrix to solve **A**_**d**_ ≈ **FB**. See also the second item in Formula 2.

Nevertheless, the standard linear regression fails to solve the linear regression model between **F** and **A**_**d**_ due to the following factors. First, the number of predictors *p* is significantly greater than that of observations *m*. Specifically, our dataset contains 603 drugs, of which each is represented as a 9149-dimensional Boolean vector of side effect occurrence (9149>> 603). Secondly, there is multicollinearity among *p* columns in **F** because some of side effects are highly correlated in terms of the occurrence of side effects (e.g. ‘*anaemic hypoxia*’ and ‘*placental dysplasia*’; ‘*salivary gland fistula*’ and ‘*alveolitis necrotising*’).

Multivariate Partial Least-Squares Regression (PLSR) is able to overcome this obstacle by mapping both the predictor and the response to a new latent space. Its way to find a linear regression model can be analogue to principal components analysis. Thus, we leverage PLSR to solve the second item in Formula 2. We adopt SIMPLS algorithm to obtain the general underlying model of PLSR since it has only one parameter (i.e. the number of latent factors) to be tuned.

### Cross validation

K-fold Cross-validation (K-CV) is one of standard approaches to evaluate the performance of algorithms in machine learning. As former approaches mentioned [19, 22–25], K-CV should be elaborated to avoid over-optimistic results in the case of predicting potential DDIs for new drugs(having no known interaction). Thus, we design two K-CV schemes, CV1 and CV2, when assessing DDI prediction tasks, which are denoted as T_1_ and T_2_ respectively (see also Fig. 1).

CV1 assesses the prediction that new drugs interact with known drugs (T_1_), while CV2 assesses the prediction that some new drugs interact with other new drugs (T_2_). Both CV1 and CV2 have several same steps as those in the regular K-CV. The whole dataset of drugs is randomly partitioned into K subsets, of which each contains an approximately equal number of drugs. The drugs in one of K subsets are chosen as the testing drugs and all the drugs in the remaining K-1 subsets are taken as the training drugs.

Remarkably, both CV1 and CV2 regard drug pairs but not drugs as samples. They have in common is that the drug pairs consisting of only the training drugs are selected as the training samples, while their difference is the selection of the testing samples.

- In each round of CV1, the testing samples are the drug pairs between the testing drugs and the training drugs, because their labels should be blind to the training phase. Such a selection of the testing samples reflects that the testing drugs (imitating new drugs) have no prior interaction with any of the training drugs. CV1 repeats the training and the testing K times by taking the drugs in each subset as the testing drugs in turn, and it averages the predicting performances in K rounds as its final performance.
- In each round of CV2, the testing samples are only the drug pairs among the testing drugs and their labels are blind to the training phase as well. Especially, the drug pairs between the testing drugs and the training drugs are rejected in the training phase, because they contain the information about the testing samples in CV2 and also discarded in the testing phase because they are the testing samples in CV1. Each round of CV2 take two subsets of drugs to label the testing drug pairs at one time. When these two subsets are same, these are K cases, which are complementary to those of CV1. When they are different to each other, there are K× (K-1)/2 cases. Totally, CV2 contains K + K× (K-1)/2 = K×(K + 1)/2 rounds and its final performance is also the average of the predicting performance of these rounds.

**Fig. 3:**
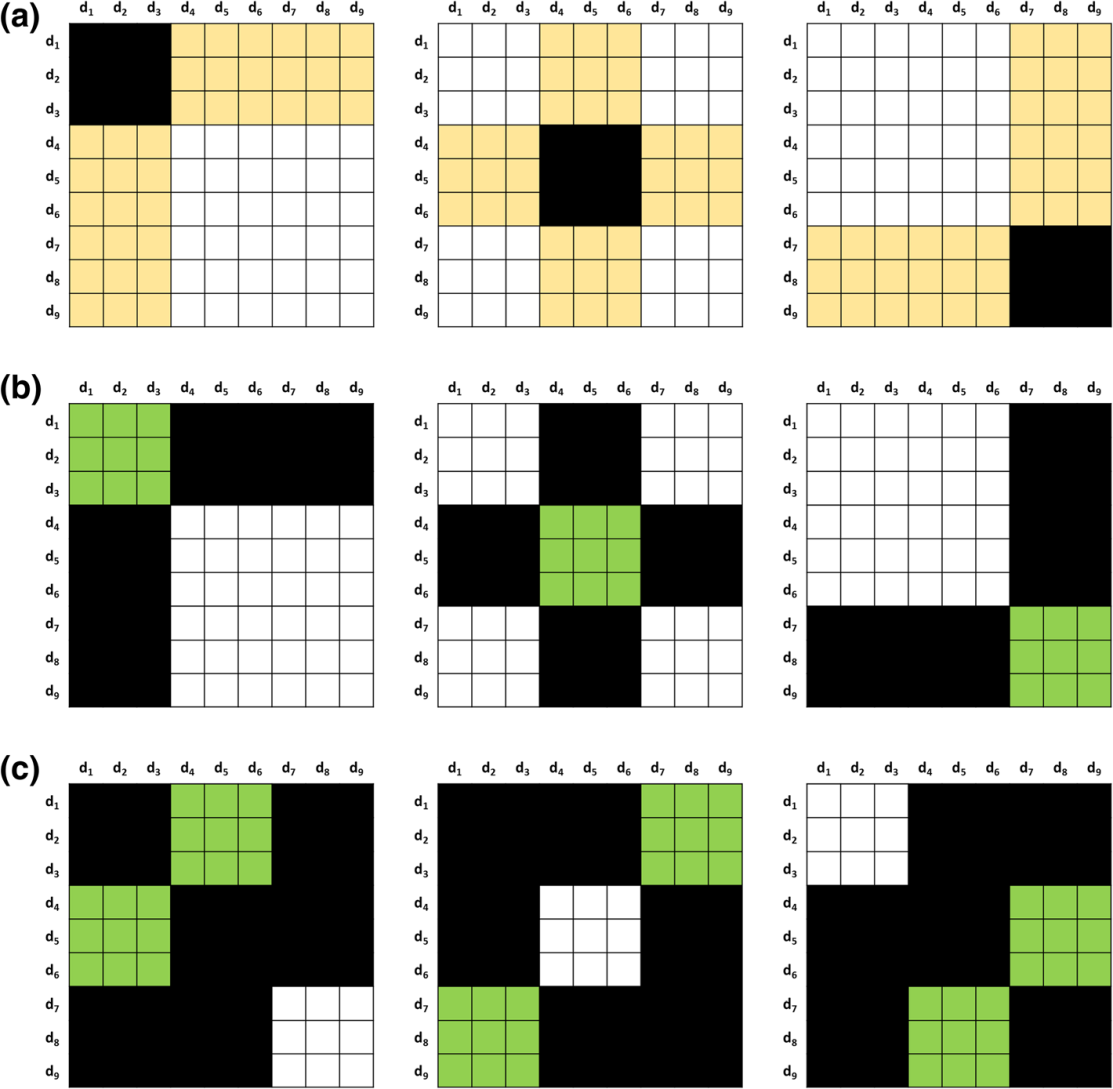
Illustration of K-CV schemes. The 9 × 9 DDI adjacent matrix is represented by a table with 9 × 9 cells. It is split into different blocks, which account for training, testing and discard parts, and are filled with different colors accordingly. All the entries in the white block are used to train the TMFUF, the entries in the yellow and the green block denote the testing entries in T1 and T_2_ respectively, and the entries in the black region are blind to the training and testing. Three subfigures show different 3-CV schemes: **a** the sampling in CV1, **b** the within-group sampling in CV2, and **c** the between-group sampling in CV2

To illustrate both CV1 and CV2, we show a toy case containing nine drugs (Fig. 3). To perform the cross validation, they’re first randomly shuffled and renumbered in ascending order. Then we perform the sampling for 3-fold cross validation sequentially.

In CV1 (Fig. 3a), we split the drugs into three exclusive groups, of which each is sampled as the testing drugs and the remaining two groups are sampled as the training drugs in turn. Consequently, CV1 performs ${C}_K^1=K=3$ rounds of CV. For example, in the second round of CV1, there are six training drugs (labeled as *d*_1_, *d*_2_, *d*_3_, *d*_7_, *d*_8_ and *d*_9_) and three testing drugs (labeled as *d*_4_, *d*_5_ and *d*_6_). A similar separation of training and testing drugs can be found in two other rounds of CV1. In Fig. 3a, the training interactions among the training drugs are denoted by white cells and used to build the predicting model, while the testing interactions between the set of the testing drugs and the set of the training drugs are denoted by yellow cells in DDI adjacent matrix. Black cells are blind to both training and testing in CV1. Totally, CV1 contains three rounds of testing.

Being stricter than CV1, CV2 contains two kinds of drug sampling, named within-group and between-group samplings. In the within-group sampling of CV2 (Fig. 3b), we also split the drugs into three groups, of which each is sampled as the testing drugs and the other two groups are sampled as the training drugs in turn. Likewise, the within-group sampling performs ${C}_K^1=K=3$ rounds of CV. Until here, the procedure is exactly same as that of CV1. However, their difference is what the testing interactions are, because this sampling simulates the scenario of predicting DDI among a set of newly given drugs. Thus, the within-group sampling takes the interactions within the testing group of drugs as the testing interactions (green cells in Fig. 3b), and the interactions among the training drugs as the training interactions (white cells in Fig. 3b). Meanwhile, the interactions between the set of the training drugs and the set of the testing drugs (black cells in Fig. 3b) are blind to both training and testing in the within-group sampling. Obviously, the within-group sampling of CV2 can be regarded as the complement of the sampling of CV1.

The between-group sampling provides a stricter way that the within-group sampling. It simulates the scenario of predicting DDI between two different sets of newly given drugs, of which any has no known interactions. In each round of the between-group sampling, two different groups are selected as the testing set and the other group of drugs is selected as the training set. Consequently, because of the symmetry of DDI adjacent matrix, the between-group sampling performs ${C}_K^2=K\left(K-1\right)/2=3$ rounds of CV. In the round, the testing interactions are only those between two testing groups of drugs (green cells in Fig. 3c) and the training interactions are only those among the training drugs (white cells in Fig. 3c). Remarkably, the interactions between the drugs in any of the testing groups and the training drugs (black cells in Fig. 3c) and the interactions among the drugs in any of the testing groups (black cells in Fig. 3c) are blind to both training and testing in the between-group sampling of CV2. Therefore, CV2 contains K×(K + 1)/2 = 6 rounds of testing in total.

To sum up, CV1 accounts for the screening scenario that we’re asked to infer how possibly a newly given drug interacts with one or more known drugs. It is particularly useful before we enlarge existing multi-drug prescriptions by appending new drugs.

While CV2 accounts for another screening scenario that we are required to determine how possibly a newly-given drug interacts with another new one. It is definitely helpful when the need to make novel multi-drug prescriptions arises. In addition, it is one of the most crucial steps towards understanding and revealing how DDIs form.

Receiver operating characteristic (ROC) curve and precision-recall (PR) curve are two of popular approaches to illustrate the performance of computational approaches in binary classification [26]. In addition, both the area under ROC curve (AUC) and the area under PR curve (AUPR) are usually adopted to measure the performance of binary classification or prediction [26].

The prediction of conventional DDIs is a typical binary prediction, in which interactions and non-interaction are labeled as positive and negative samples respectively. Thus, we can direct draw AUC and PR curves for the prediction and measure its performance with both AUC and AUPR by comparing the predicted scores of positive samples with those of negative samples.

To draw ROC and PR curves for the prediction of comprehensive DDIs, we reverse the labels of degressive DDIs and their predicting scores. Degressive DDIs are first labeled as positive samples. Then, their new scores are generated by the minus of their original predicted scores. After that, united with enhancive DDIs, they are treated as positive samples. Last, the same procedure as that of conventional DDIs is adopted to measure the prediction performance of comprehensive DDIs.

## Results and discussion

### Preparation and parameter tuning

When performing when performing CV1 for T_1_ or CV2 for T_2_, we set K = 10 such that CV1 and CV2 contain 10 and 55 rounds of the training-testing phases respectively. To achieve a statistical significance, we generated the partition of the training of the testing drugs under 50 different random seeds, and repeated CV1 and CV2 50 times accordingly. We reported the average performance over 50 repetitions of CV as the final evaluation of the prediction.

**Fig. 4:**
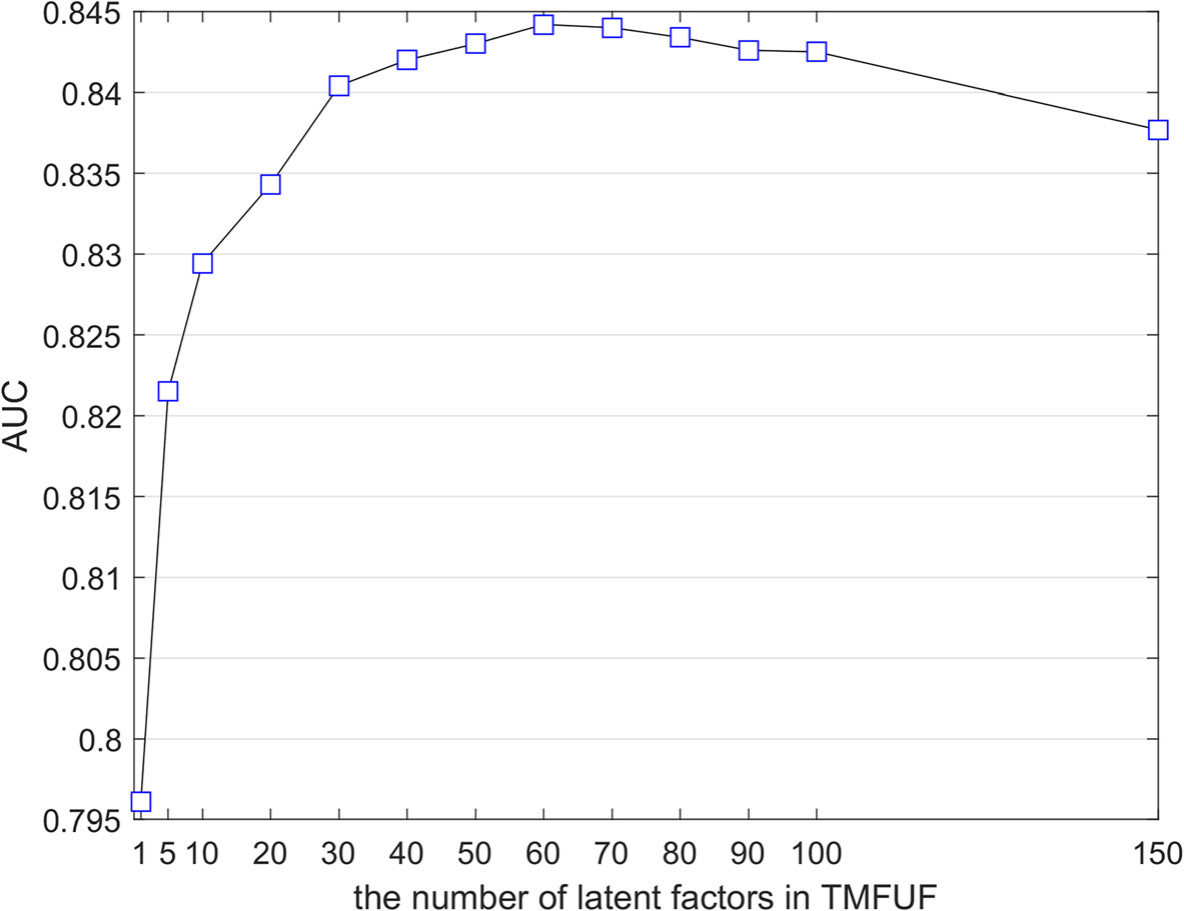
Illustration of Parameter Tuning. The number of the latent factors is tuned by a fixed list {1,5,10,20,30,40,50,60,70,80,90,100,150}. The final number is determined by the value corresponding to the maximum AUC

The number of latent factors (denoted as L in PLSR) is the only one tunable parameter in TMFUF. To obtain its best value, we adopted a simple way that performs a series of binary DDI predictions under 10CV in task T_1_ along with different values of L and measured their performance by AUC. A fixed list {1,5,10,20,30,40,50,60,70,80,90,100,150} was given for tuning L. Different values of L give different predicting results. The value (L = 60) corresponding to the maximum AUC was selected out as the best value of L (Fig. 4) and further applied in the subsequent experiments.

### Comparison with state-of-the-art

In order to validate the effectiveness of our TMFUF, we first compared it with two recent works, Naïve similarity-based approach [14] and label propagation-based approach [8], in the scenario of T_1_ with respect to conventional binary DDIs. The former approach infers how likely a newly given drug interacts with one known drug of interest by the summation of its similarities to those drugs interacting with the same drug of interest as well. The latter approach directly leverages Label Propagation (a semi-supervised classifier) to infer potential DDIs by regarding the binary DDI network as a binary label network.

In the binary prediction of DDIs, we generated the adjacent matrix of binary DDIs by simply turning “-1”s into “+ 1” in that of comprehensive DDIs. The results demonstrate that TMFUF exceeds these two state-of-the-art approaches significantly with ~ 7% and ~ 20% improvements of AUC and AUPR respectively (Table 2).

**Table 2:** Comparison with state-of-the-art methods in the scenario of T1 with respect to binary DDIs

Method	AUC	AUPR
Naïve Similarity [14]	0.759 ± 0.001	0.302 ± 0.002
Label Propagation [8]	0.774 ± 0.001	0.326 ± 0.002
TMFUF	**0.842** ± 0.002	**0.526** ± 0.006

The best results are highlighted in boldface

**Fig. 5:**
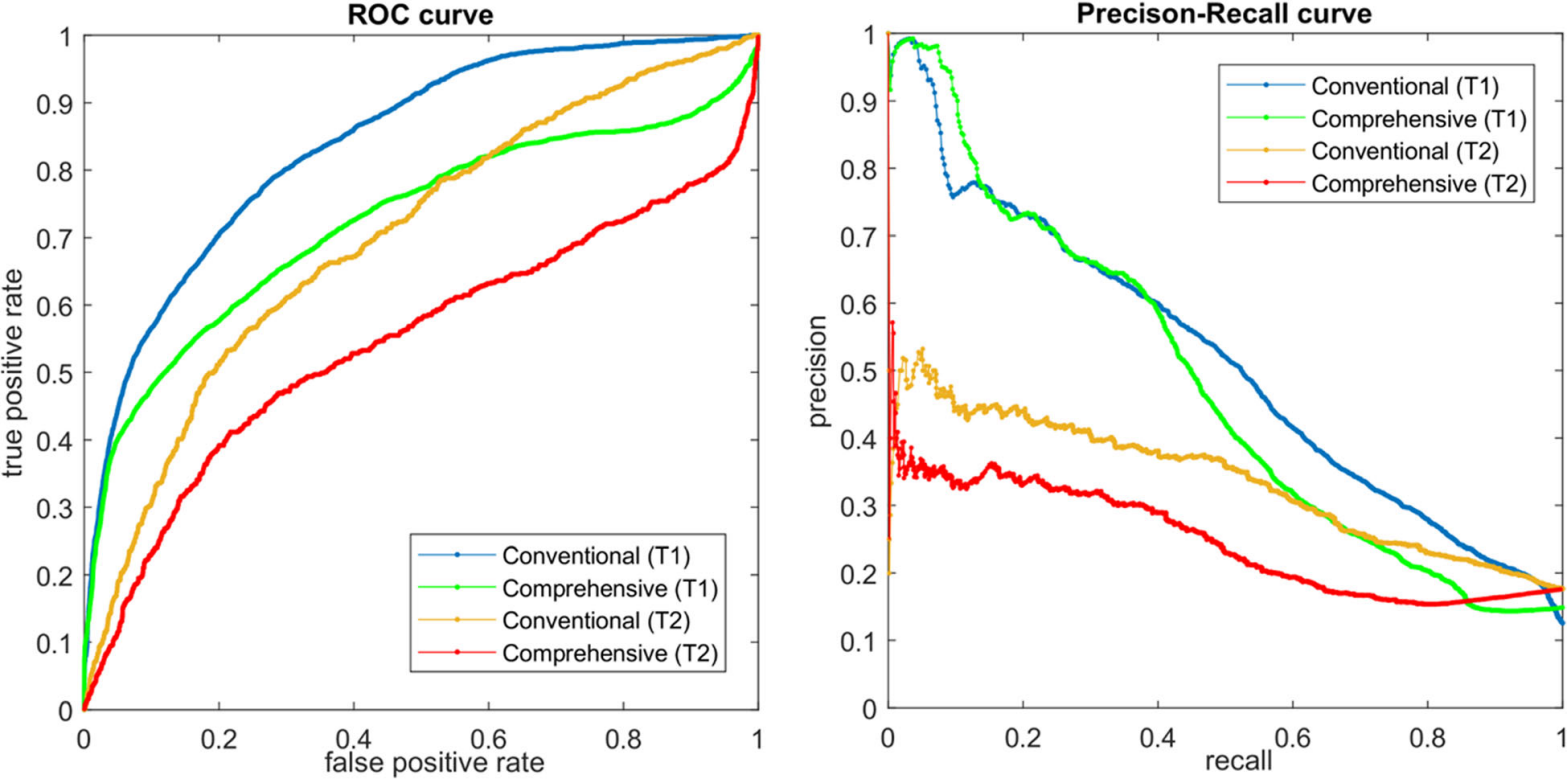
ROC and PR curves in four different scenarios with respect to conventional and comprehensive DDI prediction over two prediction tasks involving new drugs (T_1_ and T_2_)

Furthermore, we noted that these approaches were only designed for the conventional prediction of binary DDIs in the scenario of T_1_. Therefore, we also demonstrated the ability of TMFUF over four predicting scenarios with respect to two pharmacological changes caused by DDI and two screening needs for multi-drug treatments. The results are listed in Table 3 and the corresponding ROC curves are shown in Fig. 5. It can be observed that the performance of comprehensive prediction is worse than that of conventional binary prediction. For example, in task T_1_, the comprehensive prediction shows 73.3% AUC and 48.3% AUPR, while the conventional prediction shows 84.2% and 52.6% respectively. In addition, it is found that T_2_ is a more difficult task than T_1_. Compared with T_1_, T_2_ generally exhibits ~ 15% and ~ 22% degradation in terms of both AUC and AUPR.

**Table 3:** Predicting Performance of TMFUF under Different Scenarioes

Task	AUC		AUPR	
	Conventional	Comprehensive	Conventional	Comprehensive
T1	0.842 ± 0.002	0.733 ± 0.004	0.526 ± 0.006	0.483 ± 0.007
T2	0.702 ± 0.004	0.577 ± 0.005	0.303 ± 0.005	0.246 ± 0.005

### Significant feature pairs

**Fig. 6:**
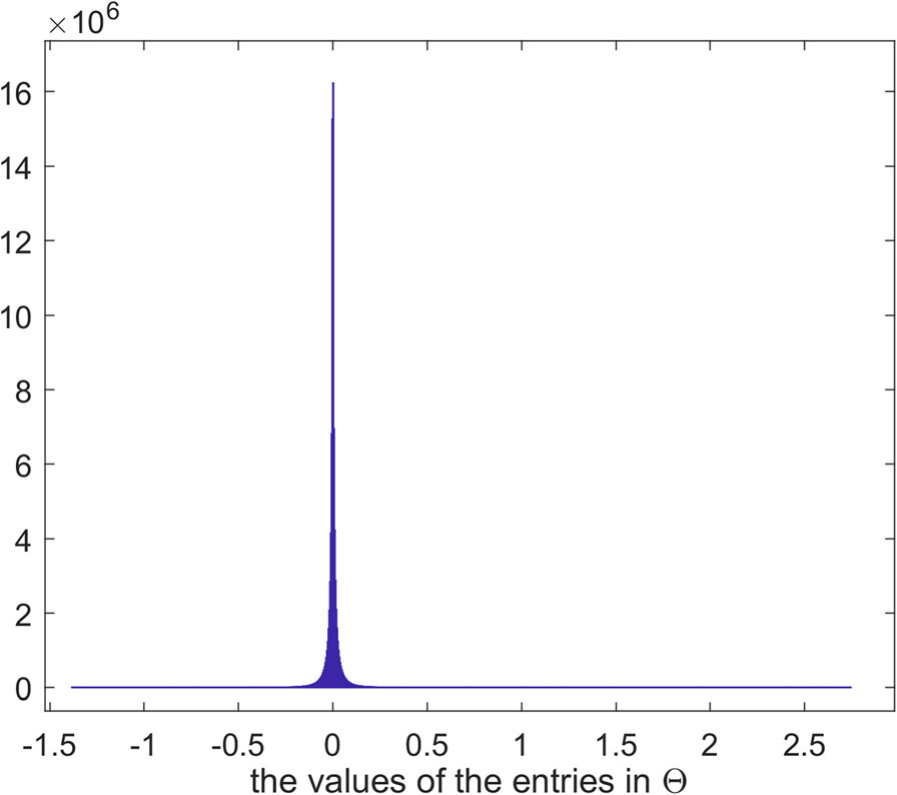
Distribution of the values of the entries in the symmetric projection matrix

According to Formula (1), we achieved the symmetric projection matrix **Θ**, which bridges drugs’ features and their interactions with the trivial reconstructed error 29.65 in terms of Frobenius Norm among 181,503 drug pairs. Especially, **Θ** is able to provide the indication of significant pairs of side effect entries. Because the number of feature entries is large, the values of the entries in **Θ** would be very small. To avoid the computational errors, we multiplied A by 1000 before solving **Θ**. The histogram of the entry values shows that very most of them are near to ZERO (Fig. 6). Therefore, we defined the entries, whose absolute values are greater than 1, as Significant Entries.

The subscripts of those significant entries correspond to the indices of features. Positive and negative significant entries indicate enhancive DDIs and degressive DDIs respectively. After sorting the entries by their absolute values, we obtained a set of significant pairs of side effects. Top 10 significant pairs for enhancive DDIs and degressive DDIs are listed in Tables 4 and 5 respectively.

**Table 4:** Top 10 Significant Pairs for Enhancive DDIs

Rank	Side Effect 1	Side Effect 2
1	‘multiple drug overdose’	‘overdose’
2	‘blood sodium’	‘bradycardia’
3	‘blood sodium’	‘overdose’
4	‘accelerated hypertension’	‘diarrhea’
5	‘glucose intolerance’	‘overdose’
6	‘diabetic neuropathy’	‘overdose’
7	‘dependence’	‘overdose’
8	‘blood sodium’	‘multiple drug overdose’
9	‘bradycardia’	‘overdose’
10	‘blood sodium’	‘drug interaction’

**Table 5:** Top 10 Significant Pairs for Degressive DDIs

Rank	Side Effect 1	Side Effect 2
1	‘accelerated hypertension’	‘hypotonia’
2	‘burning sensation mucosal’	‘overdose’
3	‘accelerated hypertension’	‘overdose’
4	‘accelerated hypertension’	‘convulsion’
5	‘accelerated hypertension’	‘diabetes mellitus inadequate control’
6	‘accelerated hypertension’	‘neuroleptic malignant syndrome’
7	‘accelerated hypertension’	‘dependence’
8	‘accelerated hypertension’	‘cholecystitis chronic’
9	‘gastrointestinal disorder’	‘overdose’
10	‘abuse’	‘accelerated hypertension’

There are several observations about these significant side effects. According to the frequency of their occurrences, the dose-related side effects and the cardiovascular system-related side effects are two types of the most significant factors in overall DDIs. The former includes ‘overdose’, ‘multiple drug overdose’ and ‘dependence’. The latter includes ‘accelerated hypertension’, ‘blood sodium’, and ‘bradycardia’. Especially, ‘overdose’ is dominant in enhancive DDIs while ‘accelerated hypertension’ is dominant in degressive DDIs. Thus, we believe that both the control of safe dosage and the adverse effects on the cardiovascular system are two important factors, which should be considered when screening DDIs in the clinic.

## Conclusions

There is a need to screen DDIs before multi-drugs prescriptions are made. Current computational approaches can rapidly deduce potential DDI candidates among a large number of drug pairs with a low cost. Nevertheless, they have two weak points. First, they are just appropriate for predicting binary DDIs, but incapable for predicting comprehensive DDIs, which increase or decrease the pharmacological behaviors of the interacting drugs in vivo. Secondly, although they are able to predict whether a new drug (having no approved interactions) interact with known drugs (have approved interactions), but cannot predict whether or not a new drug interacts with another.

To address the issues about DDIs types and screening scenarios, we have proposed a novel approaches (TMFUF) for predicting DDIs. TMFUF presents a unified solution for the prediction of both conventional binary DDIs and comprehensive DDIs under different screening scenarios. It is not only useful before we enlarge existing multi-drug prescriptions by appending new drugs, but also helpful when we need to make novel multi-drug prescriptions consisting of only new drugs. The superiority of TMFUF is demonstrated in the prediction of conventional binary and comprehensive DDIs under two screening scenarios respectively. What’s more, TMFUF is able to uncover significant feature pairs, which contribute to enhancive and degressive DDIs. In the future, we will integrate other features, (e.g. chemical structures, drug-binding proteins) into the framework of TMFUF to achieve better DDI prediction, such that it is hopeful to reveal the underlying mechanism of how DDIs form.

## Abbreviations

AUC: The area under the receiver operating characteristic curve
AUPR: The area under precision-recall curve
CV: Cross-validation
DDI: Drug-drug interaction
PLSR: Partial Least-Squares Regression
TMFUF: Triple Matrix Factorization-based Unified Framework
